# Policy recommendations for addressing privacy challenges associated with cell-based research and interventions

**DOI:** 10.1186/1472-6939-15-7

**Published:** 2014-02-03

**Authors:** Ubaka Ogbogu, Sarah Burningham, Adam Ollenberger, Kathryn Calder, Li Du, Khaled El Emam, Robyn Hyde-Lay, Rosario Isasi, Yann Joly, Ian Kerr, Bradley Malin, Michael McDonald, Steven Penney, Gayle Piat, Denis-Claude Roy, Jeremy Sugarman, Suzanne Vercauteren, Griet Verhenneman, Lori West, Timothy Caulfield

**Affiliations:** 1 Faculties of Law and Pharmacy & Pharmaceutical Sciences, University of Alberta, 116 Street and 85 Avenue, Edmonton T6G 2R3, Canada; 2 Health Law Institute, Faculty of Law, University of Alberta, 116 Street and 85 Avenue, Edmonton T6G 2R3, Canada; 3 cbcf Tumor Bank/Alberta Cancer Research Biorepository, Cross Cancer Institute, Rm 2312, 11560 University Avenue, Edmonton T6G 1Z2, Canada; 4 Faculty of Medicine, University of Ottawa, CPCR, 401 Smyth Road, Ottawa K1H 8 L1, Canada; 5 Centre of Genomics and Policy, Faculty of Medicine, and Department of Human Genetics, McGill University, 740, avenue Dr. Penfield, suite 5206, Montreal H3A 0G1, Canada; 6 Centre of Genomics and Policy, Department of Human Genetics, McGill University, 740, avenue Dr. Penfield, suite 5206, Montreal H3A 0G1, Canada; 7 Faculties of Law and Medicine, Department of Philosophy and School of Information Studies, University of Ottawa, 75 Laurier Avenue East, Ottawa K1N 6 N5, Canada; 8 Schools of Medicine and Engineering, Vanderbilt University, 2525 West End Avenue, Nashville 37203, USA; 9 W. Maurice Young Center for Applied Ethics, University of British Columbia, 2329 West Mall, Vancouver V6T 1Z4, Canada; 10 Faculty of Law, University of Alberta, 116 Street and 85 Avenue, Edmonton T6G 2R3, Canada; 11 Cell and Tissue Innovative Research Centre, University of Alberta, 116 Street and 85 Avenue, Edmonton T6G 2R3, Canada; 12 Centre de recherche Hôpital Maisonneuve-Rosemont, and Faculty of Medicine, University of Montreal, 5415 L’Assomption blvd, Montreal Quebec H1T 2M4, Canada; 13 Berman Institute of Bioethics and Department of Medicine, John Hopkins University, Baltimore Maryland 21205, USA; 14 Faculty of Medicine, University of British Columbia, 2329 West Mall, Vancouver V6T 1Z4, Canada; 15 University of Leuven, Oude Markt 13, Leuven 3000, Belgium; 16 Alberta Transplant Institute, Faculty of Medicine, University of Alberta, 116 Street and 85 Avenue, Edmonton T6G 2R3, Canada; 17 Faculty of Law and School of Public Health, University of Alberta, 116 Street and 85 Avenue, Edmonton T6G 2R3, Canada

## Abstract

**Background:**

The increased use of human biological material for cell-based research and clinical interventions poses risks to the privacy of patients and donors, including the possibility of re-identification of individuals from anonymized cell lines and associated genetic data. These risks will increase as technologies and databases used for re-identification become affordable and more sophisticated. Policies that require ongoing linkage of cell lines to donors’ clinical information for research and regulatory purposes, and existing practices that limit research participants’ ability to control what is done with their genetic data, amplify the privacy concerns.

**Discussion:**

To date, the privacy issues associated with cell-based research and interventions have not received much attention in the academic and policymaking contexts. This paper, arising out of a multi-disciplinary workshop, aims to rectify this by outlining the issues, proposing novel governance strategies and policy recommendations, and identifying areas where further evidence is required to make sound policy decisions. The authors of this paper take the position that existing rules and norms can be reasonably extended to address privacy risks in this context without compromising emerging developments in the research environment, and that exceptions from such rules should be justified using a case-by-case approach. In developing new policies, the broader framework of regulations governing cell-based research and related areas must be taken into account, as well as the views of impacted groups, including scientists, research participants and the general public.

**Summary:**

This paper outlines deliberations at a policy development workshop focusing on privacy challenges associated with cell-based research and interventions. The paper provides an overview of these challenges, followed by a discussion of key themes and recommendations that emerged from discussions at the workshop. The paper concludes that privacy risks associated with cell-based research and interventions should be addressed through evidence-based policy reforms that account for both well-established legal and ethical norms and current knowledge about actual or anticipated harms. The authors also call for research studies that identify and address gaps in understanding of privacy risks.

## Background

Uses of human biological materials for cell-based research and interventions have re-ignited persistent worries regarding the protection of genetic privacy in an era where openness, sharing, and access to affordable and accessible genetic testing technologies are increasingly commonplace. While the privacy challenges associated with cell-based research and interventions are by no means unique, they have become more evident in light of the considerable public interest and scientific excitement surrounding ground-breaking recent discoveries in the field, such as induced pluripotent stem cells (iPSCs) [1,2], somatic-cell nuclear transfer (SCNT) derived human embryonic stem cells (hESCs) [3] and triploid human embryonic stem cells (hESCs) [4]. In this article, we examine and offer recommendations for addressing these privacy challenges through the lens of cell-based research and interventions, while recognizing that the derivation and sharing of stem cell lines are a critical part of good scientific practice [5], and that the privacy challenges discussed here are engaged equally (or perhaps more markedly) in other contexts, such as in relation to genetic research and biobank datasets. Indeed, our discussion of the stem cell context will necessarily canvass and draw upon the academic literature on privacy issues facing genetic research.

In the context of cell-based research and interventions, a specific concern relates to potential privacy risks surrounding research uses of iPSCs. There is emerging scientific consensus that these stem cell lines retain substantial genetic characteristics of the parent/donor somatic cell or tissue [6,7]. Accordingly, an individual could be re-identified from anonymized or anonymous genetic data derived from such cell lines. Moreover, in most cases, cell lines will be linked to the donor’s clinical information for both research and regulatory purposes [8]. Insecure handling or misuse of these lines and associated clinical information could also result in disclosure of personal information to unauthorized parties. The highly collaborative nature of cell therapy research and the transnational movement of stem cell lines and associated health information reinforce privacy concerns, and have generated calls for policy intervention [9-11]. Privacy concerns, among other ethical and legal issues associated with cross-jurisdictional transfer of stem cell lines, also suggest a need for harmonization of policy responses across jurisdictions [12-15]. Indeed, it has been observed that conceptual and logistical impediments to international sharing of biological resources can be overcome by harmonizing privacy standards through a continuing process that fosters the interplay of different national viewpoints [16].

Furthermore, recent studies have demonstrated the possibility of re-identifying research participants from anonymized genetic data [17-19] by linking such data with freely available information in the public domain, such as familial database records, and demographic information obtained through Internet searches [10,18,20-22]. However, these re-identification studies currently require highly sophisticated technical ability and technological resources, and involve complex and specialized processes, with very limited success rates [23]. Also, institutional data use policies may preclude or impose stringent conditions on re-identification of research participants from anonymized genetic data or other health information. While it is possible that re-identification could become easier or more successful with advances in data linkage technologies, and proliferation of reference databases (including genealogy websites, genome-phenome data banks, and linked electronic medical records) [18,22,24,25], the potential risks of re-identification are presently neither manifest nor pressing in magnitude or feasibility [26]. That said, the potential for re-identification has generated significant policy and media attention and scrutiny [27-34].

It has been suggested that re-identification may cause a variety of harms, including harms to donors’ privacy interests [9,10], the possibility of genetic discrimination in the context of employment, health care, and life and medical insurance [35-37], and inappropriate disclosure of stigmatizing, embarrassing or incriminating genetic information [10,35,38]. Also, unauthorized re-identification of anonymous research participants could undermine public trust in genetic research and result in public reluctance to donate biological material for genomics research [39,40]. However, there is presently little evidence to support fears that these harms will materialize [21,41,42]. Genetic discrimination in insurance, for example, is uncommon because the predictive ability of genetic testing is limited, and most of the information that would arise is already disclosed through evaluation of family and medical history [36,42-44].

The foregoing privacy concerns are made more sensitive by emerging practices that challenge well-established legal and ethical norms. For instance, consent models, such as broad consent—which enable donors to consent to prospective, as-yet-unknown research uses of their donated materials—are increasingly common in genomics and related research contexts [45-47]. Likewise, an increasing number of policy instruments limit the right to withdraw consent to the use of donated biological materials to a time before the materials are used for research or a stem cell line is created [45,48-53]. These practices remain controversial and have generated significant discussion in the academic community [52-56]. In many jurisdictions, including Canada, Australia, the U.S. and the E.U., voluntary informed consent to identified or specific research studies is required by applicable policies [57-62]. However, research ethics committees (RECs) can approve studies that depart from this rule, on a case-by-case basis, but only if there is minimal risk to participants and the failure to obtain consent will not adversely affect participant welfare, or if it would be impossible or impractical to carry out the research without prior consent from participants [57-59].

Given the significant public interest in cell-based research and interventions, privacy is likely to be a hot area of policy debate. However, to date, there have been few, if any, attempts to examine the privacy issues arising in this context, or to formulate proactive evidence-based policy guidance to address associated risks. To this end, and under the auspices of the Office of the Privacy Commissioner of Canada Contributions Program, we convened a workshop to facilitate focused scholarly and policy reflection and analysis on the privacy risks and issues associated with cell-based research and interventions. Workshop participants consisted of the authors of this paper, and represent a multi-institutional, multidisciplinary group of legal scholars, bioethicists, privacy experts, data security experts, bioinformaticians, stem cell scientists, and trainees in all these areas. Using a workshop format we have successfully employed in the past to generate debate and consensus on policy recommendations [8,63,64], participants presented on and discussed the following topics: cell-based research and interventions, current governance regimes and associated challenges, data security and re-identification studies, privacy and open access, and consent requirements. Following the presentations, recommendations formulated by the workshop conveners (Ogbogu, Caulfield and Burningham) were presented for deliberation and revision. In the next section, we outline key themes and specific policy recommendations that emerged from the discussions at the workshop.

## Discussion

### Theme 1: Re-identification risk is a moving target

Recent research studies have demonstrated the possibility of successful re-identification of de-identified genetic data [18,19]. While these studies raise serious questions about whether de-identification-based privacy guarantees are adequate to protect research participants against unlawful use and disclosure of their genetic information, it should be borne in mind that re-identification attacks are presently technologically rigorous and expensive, have limited success rates, and require specialized equipment and access to other health data. Re-identification attacks therefore do not currently raise a level of risk that should be met with restrictive policies, such as restrictions on open access and on sharing of genetic research data. Open access policies should be combined with acceptable use or data use agreements that prohibit re-identification and/or misuse.

The risk of re-identification may increase as technology improves and/or publicly accessible databases containing genetic information linkable to identifiable individuals become more widespread. Policies designed to prevent unauthorized re-identification should be based on evidence of actual or anticipated harm, and incorporate processes for ongoing evaluation of anticipated risk or harm.

### Theme 2: Informed consent: "The devil is in the defaults"^a^

As previously stated, many jurisdictions require researchers to inform research participants about and obtain their consent to specific research uses of their biological materials and associated genetic or other health information. This requirement is typically subject to limited exceptions and must be complied with prior to commencement of research. Participants must also be made aware of any legally or ethically sanctioned limits to exercising meaningful control over their personal health or genetic information once the research has commenced.

To ensure "a consistent floor of privacy protections" [34], p. 5, these policies should be maintained as the default in relation to uses and disclosure of genetic information and associated health data. Departure from the default rules may be warranted, but only where the public interest in the departure clearly outweighs a corresponding public interest in protecting and preserving individual privacy and autonomy. The rationale for setting aside the default rules must be clearly and specifically demonstrated, and balanced against actual evidence of consequent benefits and risks. This approach is necessarily case-specific, and should be implemented by a body or institution that is familiar with, or structured to obtain and incorporate into its deliberative and decision-making process, multiple perspectives on the research context, associated privacy challenges, participant preferences, and the risks and benefits of proposed exceptions. While it remains an open question whether or not RECs can fulfill these roles within the scope of their present mandates [65], an emerging alternative is the establishment of data access committees that are charged with the responsibility of overseeing requests or applications for research use and disclosure of personal health data, and with monitoring and responding to privacy challenges resulting from innovations in health research [26,65].

### Theme 3: Beyond re-identification risk and consent: grounding the default in a "big picture" view of policy development and analysis

There is a need to move scholarly reflection beyond discussion of re-identification risks and consent issues surrounding research involving human biological materials. To encourage a shift in focus, researchers should prioritize two other relevant areas: the broader framework of policies and regulations applicable to privacy issues in this context (such as the impact of access to information law on participant rights and researcher responsibilities), and studies of affected groups’ views, such as the views of research participants, the public, and researchers working in this area. Some work has been done in both areas, including studies of public and stakeholder opinions [66-68] and a recent analysis of Canadian judicial doctrine and its implications for participants’ rights of continued access to and control over genetic and other health information [69]. However, de-identification is still an important tool in the privacy "tool-box". Even though de-identification of cell lines may not guarantee privacy, it is one tool to employ in the construction of a privacy framework and will work in conjunction with other approaches, such as education and strengthening of governance mechanisms.

In accordance with this "big picture" approach to policy development, default rules should be broadly based on existing policy rules and norms, including privacy and access to information laws, research ethics guidelines, government reports and white papers, and non-binding policy statements issued by influential scientific or research ethics organizations [34,70-80]. Gaps and warranted exceptions should be addressed through governance mechanisms designed to balance competing public interests that arise in this research context. To facilitate cross-border research collaborations, national policies should be designed to allow for harmonization with other jurisdictions.

Lastly, affected groups’ perspectives must be taken into account in designing policy, including the views of scientists, clinicians, institutional managers and research participants. Research on missing or incomplete perspectives should be encouraged and prioritized. Specifically, these groups should be included in policy deliberations and in the actual policy-making process, in addition to more traditional "top down" approaches to public consultation such as public opinion surveys, focus groups and public commentary.

### Recommendation #1: Changes to existing policies

Existing legal and ethical policies (including privacy and access to information laws and research ethics policies) should be extended to cover research involving human biological material that contains identifiable genetic information about a research participant. No special rules or exceptions need apply. Specifically:

i. research participants must be informed of known risks of re-identification of de-identified genetic data at the time of donation and consent;

ii. researchers and research institutions should inform research participants about new risks of re-identification as they emerge;

iii. researchers and institutions seeking to use or share human biological material and/or de-identified genetic data must have policies and processes in place to monitor and respond to re-identification risks, including but not limited to controlled access mechanisms;^b^

iv. legal definitions of "personal information", "personal health information" and similar terms should be expanded to include "human biological material";

v. the term "information custodian" and other similar terms in privacy and access to information legislation should be defined to include "persons or institutions that collect, use, share or disclose human biological material or genetic information derived from such samples";

vi. institutional sharing policies should address privacy protections for associated clinical health information collected with human biological material;

vii. policymakers should seek to harmonize policies across jurisdictions, and to coordinate monitoring and enforcement processes;

viii. institutions should work out inter-institutional arrangements to deal with privacy issues either through delegated or centralized review; and

ix. privacy regulators should establish mechanisms to monitor technological developments and review and update best practices in relation to privacy risks attending to research uses of human biological materials.

### Recommendation #2: Changes to governance mechanisms

The role of RECs in privacy governance in the context of cell-based research should be clarified. At the moment, some hurdles may stand in the way of effective oversight, including the fact that RECs may lack experience in privacy matters or may exchange rigorous ethics review for bureaucratic box checking [65,81,82]. Accordingly, legislation and relevant policies should set out dedicated governance frameworks to monitor and respond to privacy challenges in the context of cell-based research. Options to consider include:

i. revising membership requirements to include mandatory representation by a privacy expert or IT security specialist; or

ii. establishing an independent "data access committee" to review research protocols that raise significant privacy concerns (perhaps on a referral basis from RECs) and to provide general guidance in response to anticipated or existing privacy challenges.

## Summary

Addressing privacy challenges and issues facing cell-based research and interventions requires collaborative reflection among and response from multiple interested parties, including scientists, privacy experts, bioethicists, legal scholars and policymakers. This paper outlines the first attempt at such an endeavour, and provides a summary of key themes and recommendations to facilitate and guide both future discussions and policymaking activities in this context. While the issues canvassed in the paper, chiefly the privacy risks surrounding ongoing linkage of stem cell lines to research participants’ genetic and clinical information, deserve scholarly and policy scrutiny, they are not necessarily unique. They must therefore be met with measured evidence-based policy reforms that account for both well-established legal and ethical norms and current knowledge about actual or anticipated harms. Research on privacy issues in this context should focus on gaps in knowledge, such as canvassing the views of persons or groups whose interests are most likely to be affected. Lastly, policy development in this context must be necessarily proactive and aimed primarily at maintaining public trust in and support for cell-based research and interventions.

## Endnotes

^a^This phrase is borrowed from Ian Kerr’s presentation at the workshop, and is referenced in his earlier editorial discussing Facebook and privacy [83].

^b^In controlled-access agreements, one party agrees to provide the other with access to specific data or material on certain conditions relating to security practices or confidentiality [84].

## Competing interests

The authors declare that they have no competing interests.

## Authors’ contributions

All authors contributed to the conception and design of the paper. UO, SB and AO drafted the manuscript. All authors contributed revisions to the manuscript. All authors read and approved the final manuscript.

## Pre-publication history

The pre-publication history for this paper can be accessed here:

http://www.biomedcentral.com/1472-6939/15/7/prepub

## References

[B1] Takahashi K Tanabe K Ohnuki M Narita M Ichisaka T Tomoda K Yamanaka S Induction of pluripotent stem cells from adult human fibroblasts by defined factors Cell 2007 131 861 872 18035408 10.1016/j.cell.2007.11.019

[B2] Yu J Vodyanik MA Smuga-Otto K Antosiewicz-Bourget J Frane JL Tian S Nie J Jonsdottir GA Ruotti V Stewart R Slukvin II Thomson JA Induced pluripotent stem cell lines derived from human somatic cells Science 2007 318 1917 1920 18029452 10.1126/science.1151526

[B3] Tachibana M Amato P Sparman M Gutierrez NM Tippner-Hedges R Ma H Kang E Fulati A Lee H-S Sritanaudomchai H Masterson K Larson J Eaton D Sadler-Fredd K Battaglia D Lee D Wu D Jensen J Patton P Gokhale S Stouffer RL Wolf D Mitalipov S Human embryonic stem cells derived by somatic cell nuclear transfer Cell 2013 153 1228 1238 23683578 10.1016/j.cell.2013.05.006 PMC3772789

[B4] Noggle S Fung HL Gore A Martinez H Satriani KC Prosser R Oum K Paull D Druckenmiller S Freeby M Greenberg E Zhang K Goland R Sauer MV Leibel RL Egli D Human oocytes reprogram somatic cells to a pluripotent state Nature 2011 478 70 75 21979046 10.1038/nature10397

[B5] Chalmers DRC Rathjen PD Rathjen J Nicol D Stem cells and regenerative medicine: From research to clinical applications J Law Med 2013 20 831 844

[B6] Pryzkhova M Stem cells: will they ever be the same? Regen Med 2013 8 97 99 23477389 10.2217/rme.13.9

[B7] Abyzov A Mariani J Palejev D Zhang Y Haney MS Tomasini L Ferrandino AF Rosenberg Belmaker LA Szekely A Wilson M Kocabas A Calixto NE Grigorenko EL Huttner A Chawarska K Weissman S Urban AE Gerstein M Vaccarino FM Somatic copy number mosaicism in human skin revealed by induced pluripotent stem cells Nature 2012 492 438 442 23160490 10.1038/nature11629 PMC3532053

[B8] Zarzeczny A Scott C Hyun I Bennett J Chandler J Chargé S Heine H Isasi R Kato K Lovell-Badge R McNagy K Pei D Rossant J Surani A Taylor PL Ogbogu U Caulfield T iPS cells: mapping the policy issues Cell 2009 139 1032 1037 20005794 10.1016/j.cell.2009.11.039

[B9] Lunshof JE Chadwick R Vorhaus DB Church GM From genetic privacy to open consent Nat Rev Genet 2008 9 406 411 18379574 10.1038/nrg2360

[B10] Craig DW Goor RM Wang Z Paschall J Ostell J Feolo M Sherry ST Manolio TA Assessing and managing risk when sharing aggregate genetic variant data Nat Rev Genet 2011 12 730 736 21921928 10.1038/nrg3067 PMC3349221

[B11] Isasi R Knoppers BM Andrews PW Bredenoord A Colman A Hin LE Hull S Kim OJ Lomax G Morris C Sipp D Stacey G Wahlstrom J Zeng F Disclosure and management of research findings in stem cell research and banking: policy statement Regen Med 2012 7 439 448 22594334 10.2217/rme.12.23

[B12] Caulfield T Zarzeczny A McCormick J Bubela T Critchley C Einsiedel E Galipeau J Harmon S Huynh M Hyun I Illes J Isasi R Joly Y Laurie G Lomax G Longstaff H McDonald M Murdoch C Ogbogu U Owen-Smith J Pattinson S Premji S von Tigerstrom B Winickoff DE The stem cell research environment: a patchwork of patchworks Stem Cell Rev Rep 2009 5 82 88 10.1007/s12015-009-9071-3 19521798

[B13] Caulfield T Zarzeczny A McCormick J Bubela T Critchley C Einsiedel E Galipeau J Harmon S Huynh M Hyun I Illes J Isasi R Joly Y Laurie G Lomax G Longstaff H McDonald M Murdoch C Ogbogu U Owen-Smith J Pattinson S Premji S von Tigerstrom B Winickoff DE International stem cell environments: a world of difference Nature Reports Stem Cells 2009 doi:10.1038/stemcells.2009.61

[B14] Isasi RM Policy interoperability in stem cell research: demystifying harmonization Stem Cell Rev Rep 2009 5 108 115 10.1007/s12015-009-9067-z 19521802

[B15] Isasi R Knoppers BM From banking to international governance: fostering innovation in stem cell research Stem Cells Intl 2011 doi:10.4061/2011/498132 10.4061/2011/498132 PMC3167189 21904557

[B16] Chadwick R Strange H Widdows H, Mullen C Harmonisation and standardisation in ethics and governance: conceptual and practical challenges The Governance of Genetic Information: Who Decides? Volume 9 2009 Cambridge: Cambridge University Press 201 213

[B17] Lin Z Owen AB Altman RB Genomic research and human subject privacy Science 2004 305 183 15247459 10.1126/science.1095019

[B18] Gymrek M McGuire AL Golan D Halperin E Erlich Y Identifying personal genomes by surname inference Science 2013 339 321 324 23329047 10.1126/science.1229566

[B19] Homer N Szelinger S Redman M Duggan D Tembe W Muehling J Pearson JV Stephan DA Nelson SF Craig DW Resolving individuals contributing trace amounts of DNA to highly complex mixtures using high-density SNP genotyping microarrays PLoS Genet 2008 4 e1000167 18769715 10.1371/journal.pgen.1000167 PMC2516199

[B20] Wjst M Caught you: threats to confidentiality due to the public release of large-scale genetic data sets BMC Med Ethics 2010 11 21 21190545 10.1186/1472-6939-11-21 PMC3022540

[B21] Malin B Loukides G Benitez K Clayton EW Identifiability in biobanks: models, measures, and mitigation strategies Hum Genet 2011 130 383 392 21739176 10.1007/s00439-011-1042-5 PMC3621020

[B22] Malin B Re-identification of familial database records AMIA Annu Symp Proc 2006 2006 524 528 17238396 PMC1839550

[B23] El Emam K Jonker E Arbuckle L Malin B A systematic review of re-identification attacks on health data PLoS One 2011 6 e28071 22164229 10.1371/journal.pone.0028071 PMC3229505

[B24] Schmidt H Callier S How anonymous is 'anonymous’? Some suggestions towards a coherent universal coding system for genetic samples J Med Ethics 2012 38 304 309 22345546 10.1136/medethics-2011-100181 PMC3390742

[B25] Greenbaum D Genomic data disclosure: time to reassess the realities Am J Bioeth 2013 13 5 47 50 23557049 10.1080/15265161.2013.776134

[B26] Joly Y Dove ES Knoppers BM Bobrow M Chalmers D Data sharing in the post-genomic world: the experience of the International Cancer Genome Consortium (ICGC) Data Access Compliance Office (DACO) PLoS Comput Biol 2012 8 e1002549 22807659 10.1371/journal.pcbi.1002549 PMC3395593

[B27] Kolata G Web Hunt for DNA Sequences Leaves Privacy Compromised 2013 New York Times http://www.nytimes.com/2013/01/18/health/search-of-dna-sequences-reveals-full-identities.html

[B28] Ferguson W A Hacked Database Prompts Debate about Genetic Privacy 2013 Scientific American http://www.scientificamerican.com/article.cfm?id=a-hacked-database-prompts

[B29] BBC News Donated Genetic Data 'Privacy Risk 2013 http://www.bbc.co.uk/news/health-21056647

[B30] Tanner A Harvard Professor re-identifies Anonymous Volunteers in DNA Study 2013 Forbes http://www.forbes.com/sites/adamtanner/2013/04/25/harvard-professor-re-identifies-anonymous-volunteers-in-dna-study/

[B31] Skloot R The Immortal Life of Henrietta Lacks, The Sequel 2013 New York Times http://www.nytimes.com/2013/03/24/opinion/sunday/the-immortal-life-of-henrietta-lacks-the-sequel.html?pagewanted=all

[B32] Coghlan A Storm Erupts Over Publishing of HeLa Genome New Scientist http://www.newscientist.com/article/dn23330-storm-erupts-over-publishing-of-hela-genome.html

[B33] Kroll D The Henrietta Lacks Genome: Consent, Trust, and Common Decency Forbes http://www.forbes.com/sites/davidkroll/2013/03/24/the-henrietta-lacks-genome-consent-trust-and-common-decency/

[B34] Presidential Commission for the Study of Bioethical Issues Privacy and Progress in Whole Genome Sequencing 2012 Washington, DC http://bioethics.gov/node/764

[B35] Lowrance WW Collins FS Identifiability in genomic research Science 2007 317 600 602 17673640 10.1126/science.1147699

[B36] Joly Y Braker M Le Huynh M Genetic discrimination in private insurance: global perspectives New Genet Soc 2010 29 351 368

[B37] Lipworth W Forsyth R Kerridge I Tissue donation to biobanks: a review of sociological studies Sociol Health Illn 2011 33 792 811 21592141 10.1111/j.1467-9566.2011.01342.x

[B38] Altman RB Clayton EW Kohane IS Malin BA Roden DM Data re-identification: societal safeguards Science 2013 339 1032 1033 23449577 10.1126/science.339.6123.1032-c PMC3740512

[B39] McGuire AL Gibbs RA No longer de-identified Science 2006 312 370 371 16627725 10.1126/science.1125339

[B40] Rodriguez LL Brooks LD Greenberg JH Green ED The complexities of genomic identifiability Science 2013 339 275 276 23329035 10.1126/science.1234593

[B41] Weil CJ Mechanic LE Green T Kinsinger C Lockhart NC Nelson SA Rodriguez LL Buccini LD NCI think tank concerning the identifiability of biospecimens and "omic" data Genet Med 2013 doi:10.1038/gim.2013.40 10.1038/gim.2013.40 PMC4097316 23579437

[B42] Joly Y Ngueng Feze I Simard J Genetic discrimination and life insurance: a systematic review of the evidence BMC Med 2013 11 25 23369270 10.1186/1741-7015-11-25 PMC3606414

[B43] Joly Y Knoppers BM Godard B Genetic information and life insurance: a 'real’ risk? Eur J Hum Genet 2003 11 561 564 12891375 10.1038/sj.ejhg.5200998

[B44] Thomas RG Genetics and insurance in the United Kingdom 1995–2010: the rise and fall of 'scientific’ discrimination New Genet Soc 2012 31 203 222

[B45] Caulfield T The biobanking quandary: getting and withdrawing consent Harvard Health Policy Rev 2011 12 21 24

[B46] Aalto-Setälä K Conklin BR Lo B Obtaining consent for future research with induced pluripotent cells: opportunities and challenges PLoS Biol 2009 7 e1000042 10.1371/journal.pbio.1000042 PMC2652391 19243222

[B47] Kapp M Biobanking human biological materials: issues surrounding the collection of samples for use in future research Pharmaceut Med 2008 22 75 84

[B48] Canadian Institutes of Health Research Updated Guidelines for Human Pluripotent Stem Cell Research 2010 http://www.cihr-irsc.gc.ca/e/42071.html

[B49] Academies N Guidelines for Human Embryonic Stem Cell Research 2005 Washington, DC: National Academies Press

[B50] Stem Cell Network Use of Human Embryos for Stem Cell Researchhttp://www.stemcellnetwork.ca/uploads/document-library/policy-papers/policy_humanembryo.pdf

[B51] International Society for Stem Cell Research Guidelines for the Conduct of Human Embryonic Stem Cell Research (December 2006) http://www.isscr.org/home/publications/guide-clintrans

[B52] Hoffman B Broadening consent–and diluting ethics? J Med Ethics 2009 35 125 129 19181887 10.1136/jme.2008.024851

[B53] Caulfield T Ogbogu U Isasi RM Informed consent in embryonic stem cell research: are we following basic principles? CMAJ 2007 176 1722 1725 17548387 10.1503/cmaj.061675 PMC1877848

[B54] Master Z Nelson E Murdoch B Caulfield T Biobanks, consent and claims of consensus Nat Methods 2012 9 885 888 22936169 10.1038/nmeth.2142

[B55] Eriksson S Helgesson G Potential harms, anonymization, and the right to withdraw consent to biobank research Eur J Hum Genet 2005 13 1071 1076 15986039 10.1038/sj.ejhg.5201458

[B56] Hug K Hermerén G Johansson M Withdrawal from biobank research: considerations and the way forward Stem Cell Rev 2012 8 1056 1065 22836808 10.1007/s12015-012-9399-y

[B57] Canadian Institutes of Health Research, Natural Sciences and Engineering Research Council of Canada, and Social Sciences and Humanities Research Council of Canada Tri-Council Policy Statement: Ethical Conduct for Research Involving Humans http://www.ethics.gc.ca/pdf/eng/tcps2/TCPS_2_FINAL_Web.pdf

[B58] Protection of Human Subjects (Common Rule), 45 CFR §46.116 http://www.hhs.gov/ohrp/humansubjects/guidance/45cfr46.html#46.116

[B59] National Health and Medical Research Council, Australian Research Council, Australian Vice-Chancellors’ Committee National Statement on Ethical Conduct in Human Researchhttp://www.nhmrc.gov.au/guidelines/publications/e72

[B60] Directives EC, Directive 2001/20/EC of the European Parliament and of the Council of 4 April 2001 on the Approximation of the Laws, Regulations and Administrative Provisions of the Member States Relating to the Implementation of Good Clinical Practice in the Conduct of Clinical Trials on Medicinal Products for Human Use, [2001] OJ L 121/34 http://eur-lex.europa.eu/LexUriServ/LexUriServ.do?uri=OJ:L:2001:121:0034:0044:en:PDF 16276663

[B61] European Commission–CORDIS–Seventh Framework Programme (FP7) Guidance for Applicants: Informed Consenthttp://ec.europa.eu/research/participants/data/ref/fp7/89807/informed-consent_en.pdf

[B62] UK Department of Health Reference Guide to Consent for Examination or Treatment (2nd ed) https://www.gov.uk/government/publications/reference-guide-to-consent-for-examination-or-treatment-second-edition

[B63] Caulfield T McGuire AL Cho M Buchanan JA Burgess MM Danilczyk U Diaz CM Fryer-Edwards K Green SK Hodosh MA Juengst ET Kaye J Kedes L Knoppers BM Lemmens T Meslin EM Murphy J Nussbaum RL Otlowski M Pullman D Ray PN Sugarman J Timmons M Research ethics recommendations for whole-genome research: consensus statement PLoS Biol 2008 6 e73 18366258 10.1371/journal.pbio.0060073 PMC2270329

[B64] Caulfield T Ogbogu U Nelson E Einsiedel E Knoppers B McDonald M Brunger F Downey R Fernando K Galipeau J Geransar R Griener G Hyun I Isasi R Kardel M Knowles L Kucic T Lotjonen S Lyall D Magnus D Mathews DJ Nisbet M Nisker J Pare G Pattinson S Pullman D Rudnicki M Williams-Jones B Zimmerman S Stem cell research ethics: consensus statement on emerging issues J Obstet Gynaecol Can 2007 29 843 848 17915069 10.1016/s1701-2163(16)32632-9

[B65] Ogbogu U Burningham S Privacy protection and genetic research: where does the public interest lie? Alberta Law Review 2014 51 3 in press

[B66] Gollust SE Gordon ES Zayac C Griffin G Christman MF Pyeritz RE Wawak L Bernhardt BA Motivations and perceptions of early adopters of personalized genomics: perspectives from research participants Public Health Genomics 2012 15 22 30 21654153 10.1159/000327296 PMC3225236

[B67] Critchley CR Public opinion and trust in scientists: the role of the research context, and the perceived motivation of stem cell researchers Public Underst Sci 2008 17 309 327 19069082 10.1177/0963662506070162

[B68] Master Z Claudio JO Rachul C Wang JC Minden MD Caulfield T Cancer patient perceptions on the ethical and legal issues related to biobanking BMC Med Genomics 2013 6 8 23497701 10.1186/1755-8794-6-8 PMC3599691

[B69] Ogbogu U Burningham S Caulfield T The right to control and access genetic research information: does *McInerney* offer a way out of the consent/withdrawal conundrum? UBC Law Review 2014 47 1 in press

[B70] National Academy of Sciences Board on Biology, and National Research Council: Privacy Issues in Biomedical and Clinical Research 2008 Washington, DC: National Academies Press

[B71] National Cancer Institute (Ed) NCI Best Practices for Biospecimen Resources http://biospecimens.cancer.gov/bestpractices/2011-NCIBestPractices.pdf.

[B72] International Cancer Genome Consortium Policy on Informed Consent, Access and Ethical Oversighthttp://icgc.org/icgc/goals-structure-policies-guidelines/e1-informed-consent-access-and-ethical-oversight

[B73] Australian Medical Association Position Statement on Genetic Testing 2012 https://ama.com.au/position-statement/genetic-testing-2012

[B74] Council for International Organizations of Medical Sciences International Ethical Guidelines for Biomedical Research Involving Human Subjectshttp://www.cioms.ch/publications/layout_guide2002.pdf 14983848

[B75] Organization for Economic Cooperation and Development OECD Guidelines on Human Biobanks and Genetic Research Databaseshttp://www.oecd.org/science/biotechnologypolicies/44054609.pdf

[B76] World Health Organization Special Programme of Research, Development and Research Training in Human Reproduction: Guideline for Obtaining Informed Consent for the Procurement and Use of Human Tissues, Cells and Fluids in Researchhttp://www.who.int/reproductivehealth/topics/ethics/human_tissue_use.pdf

[B77] Council of Europe, Committee of Ministers Recommendation Rec(2006)4 of the Committee of Ministers to member states on research on biological materials of human origin https://wcd.coe.int/ViewDoc.jsp?id=977859

[B78] Safran C Bloomrosen M Hammond WE Labkoff S Markel-Fox S Tang PC Detmer DE Toward a National Framework for the Secondary Use of Health Data: An American Medical Informatics Association White Paper J Am Med Inform Assoc 2007 14 1 9 17077452 10.1197/jamia.M2273 PMC2329823

[B79] Geissbuhler A Safran C Buchan I Bellazzi R Labkoff S Eilenberg K Leese A Richardson C Mantas J Murray P De Moor G Trustworthy reuse of health data: A transnational perspective Int J Med Inform 2012 82 1 9 23182430 10.1016/j.ijmedinf.2012.11.003

[B80] Knoppers BM Harris JR Tassé AM Budin-Ljøsne I Kaye J Deschênes M Zawati MH Towards a data sharing Code of Conduct for international genomic research Genome Med 2011 3 46 21787442 10.1186/gm262 PMC3221544

[B81] Hirtle M The governance of research involving human participants in Canada Health Law J 2003 11 137 152 15600072

[B82] McDonald M Canadian governance of health research involving human subjects: is anybody minding the store? Health Law J 2001 9 1 21 12141216

[B83] Kerr I The Devil is in the Defaults 2010 Ottawa Citizen http://www2.canada.com/ottawacitizen/story.html?id=f3fc40da-dee3-4a1d-bf05-d35301352010

[B84] Joly Y Zeps N Knoppers BM Genomic databases access agreements: legal validity and possible sanctions Hum Genet 2011 130 441 449 21706183 10.1007/s00439-011-1044-3

